# Trinitrotoluene and mandarin peels selectively affect lignin-modifying enzyme production in white-rot basidiomycetes

**DOI:** 10.1186/s40064-016-1895-0

**Published:** 2016-03-01

**Authors:** Eva Kachlishvili, Mikheil Asatiani, Aza Kobakhidze, Vladimir Elisashvili

**Affiliations:** Department of Plant Substrates Bioconversion, Agricultural University of Georgia, 240 David Agmashenebeli alley, 0159 Tbilisi, Georgia

**Keywords:** White-rot basidiomycetes, 2, 4, 6-Trinitrotoluene degradation, Laccase, Manganese peroxidase, Enzyme production

## Abstract

Five white-rot basidiomycetes (WRB) species have been evaluated for their potential to tolerate and to degrade 0.2 mM 2, 4, 6-trinitrotoluene (TNT) as well as to produce laccase and manganese peroxidase (MnP) in presence of this xenobiotic. The tested fungal strains produced laccase in both glycerol and mandarin peels-containing media, whereas in the glycerol-containing medium only *Cerrena unicolor* strains and *Trametes versicolor* BCC 775 secreted MnP. Replacement of glycerol by milled mandarin peels 3- to 45-fold increased laccase activity, promoted *C. unicolor* strains and *T. versicolor* MnP secretion and induced this enzyme production by *Fomes fomentarius* BCC 38 and *Funalia trogii* BCC 146. Differential response of the WRB strains to the TNT addition was observed. In particular, laccase activity of *C. unicolor* increased 2- to 3-fold in both media whereas no stimulation of the laccase production was revealed in cultivation of *F. fomentarius*. TNT practically did not affect the MnP activity. Two strains of *C. unicolor* followed by *T. versicolor* producing laccase and MnP almost completely removed 0.2 mM TNT from the synthetic medium. Increase of TNT concentration from 0 to 0.4 mM in the mandarin peels-based medium and from 0 to 0.3 mM in the glycerol-containing medium stimulated *C. unicolor* BCC 300 laccase production from 92.4 to 240.7 U/ml and from 17.1 to 48.6 U/ml, respectively. This strain has been resistant to the TNT high concentration and has ability to remove 85 % of initial 0.3 mM TNT content during 6 days of the submerged cultivation.

## Background

Contamination of soil and water by a nitroaromatic explosive 2, 4, 6-trinitrotoluene (TNT) and its metabolites toxic and mutagenic to humans, animals, plants, and microorganisms represents a worldwide environmental problem (Lewis et al. 2004; Claus 2014). There are many physical and chemical tools and approaches suggested for remediation of soils contaminated with TNT, but all of them are very expensive. Bioremediation, in particular, mycoremediation is considered as an economical and environmentally friendly approach to detoxify TNT-contaminated soils. Several studies have shown that the white-rot basidiomycetes (WRB) degrade a wide variety of recalcitrant and toxic chemicals and they are capable of the oxidative destruction and mineralization an aromatic nucleus of TNT (Fernando et al. 1990; Van Aken et al. 1997, 1999). The model organism, *Phanerochaete chrysosporium* has been the subject of intensive studies; however, growth of this fungus was inhibited with relatively low concentrations of TNT (Esteve-Núñez et al. 2001).

Evidences were gathered suggesting involvement of the wood- and litter-decaying basidiomycetes ligninolytic system in the TNT mineralization process under different nutrient starvation conditions; especially significant level of MnP is an important factor in ensuring high biodegradation rates (Fernando et al. 1990; Scheibner et al. 1997; Van Aken et al. 1997, 1999; Kim and Song 2000, 2003; Rho et al. 2001). Recently, Anasonye et al. (2015) showed that *Gymnopilus luteofolius*, *Kuehneromyces mutabilis*, and *Phanerochaete velutina* produced high amounts of manganese peroxidase (MnP) in TNT contaminated non-sterile soil. The most efficient fungus, *P. velutina* degraded 80 % of TNT during 2.5 months.

The WRB produce the nonspecific lignin-degrading system including lignin peroxidase (EC 1.11.1.14), MnP (EC 1.11.1.13), and laccase (EC 1.10.3.2) in different combinations and a number of the accessory enzymes and metabolites. Although many reports have been published about the mineralization of TNT by WRB and litter decay fungi, very scarce information is available on these enzymes synthesis in response to the TNT presence in nutrient media. Only Cheong et al. (2006) showed the increased expression of the laccase gene during early stage of TNT transformation by *Trametes versicolor* without estimation of enzyme activity. Moreover, effect of TNT on the laccase and MnP production by *Cerrena unicolor* and *T. versicolor* was elucidated, but without simultaneous determination of xenobiotic degradation (Elisashvili et al. 2010). Therefore, the main objective of this study was to evaluate a capability of six WRB belonging to different taxonomic groups to express the lignin-modifying enzymes (LME) activity in response to TNT supplementation, to tolerate and to remove comparatively high concentrations of this xenobiotic. In addition, the laccase and MnP secretion modulation by varying TNT concentration and xenobiotic elimination by *C. unicolor* BCC 300 were examined for the first time in the short-term experiments. Moreover, the significance of nutrient medium composition for the target enzymes activity expression was also assessed.

## Methods

### Organisms and inoculum preparation

The following WRB from the basidiomycete’s culture collection of the Department of Plant Substrates Bioconversion have been used in this study: *C. unicolor* BCC 300, *C. unicolor* BCC 302, *Fomes fomentarius* BCC 38, *Funalia trogii* BCC 146, *Pycnoporus coccineus* BCC 310, and *T. versicolor* BCC 775. For the identification all selected strains have been undergone to genomic DNA extraction, PCR and Internal Transcribed Spacer (ITS) sequencing. DNAs were analyzed with FlashGel™ DNA Cassettes; amplification of the ITS fragments was performed according to the CIRM-CF protocol using two universal primers ITS 1 (TCC GTA GGT GAA CCT GCG G) and ITS 4 (TCC TCC GCT TAT TGA TAT GC). Molecular identification was performed using NCBI BLAST with the counting as query sequence. Nomenclatural update was made according to the Index Fungorum (http://www.indexfungorum.org/) and MycoBank (http://fr.mycobank.org/).

The fungi are maintained on agar plates containing 1 % malt extract, 0.2 % peptone, 0.2 % yeast extract, and 2 % wheat bran and they are subcultured at regular intervals. The 1–3 weeks-old mycelium scraped from the agar surface was used for inocula preparation. The fungal inocula were prepared by growing their mycelia on a rotary shaker at 27 °C and 150 rpm in 250-ml flasks containing 100 ml of the medium appropriate for cultivation of WRB and sufficient biomass accumulation (g/l): glucose, 10; NH_4_NO_3_, 2; KH_2_PO_4_, 1; MgSO_4_·7H_2_O, 0.5; yeast extract, 2. After 7 days of cultivation the fungal biomass was homogenized in a Waring laboratory blender.

### Cultivation conditions

The submerged cultivation of fungus was conducted in the Innova 44 shaker (New Brunswick Scientific, USA) at 27 °C and 150 rpm. The homogenized mycelium (3 ml) was used to inoculate the 250-ml flasks containing 50 ml of the basal medium (g/l): ammonium tartrate, 2; KH_2_PO_4_, 1; yeast extract (Acumedia, Michigan, USA), 3; CuSO_4_·5H_2_O, 0.02; MnSO_4_, 0.01; pH 5.8. Copper and manganese have been added to the medium to stimulate, respectively, laccase and MnP production (Bertrand et al. 2013; Janusz et al. 2013). Glycerol (synthetic medium) in concentration of 10 g/l or milled to powder mandarin peels (lignocellulose-containing medium) in an amount of 40 g/l were used as growth substrates.

To evaluate the effect of TNT on the selected fungi growth and LME expression this compound was added at concentration of 0.2 mM to the synthetic or mandarin peels (MP) containing media before inoculation. Submerged cultivation of fungi was carried out during 14 days. In these experiments, 1 ml samples were taken after 5, 7, 10, and 14 days of fungi cultivation and the solids were separated by centrifugation (Eppendorf 5417R, Germany) at 10,000*g* for 5 min at 4 °C. The enzyme activities and pH were analyzed in the supernatants. In addition, some short-term experiments have been conducted in the glycerol-based medium to control of the fungus biomass accumulation and to study the initial effect of TNT concentration on the *C. unicolor* BCC 300 LME accumulation profiles. In this case, 20 ml of homogenized mycelium was inoculated in flasks containing 80 ml of basal medium with 10 g/l glycerol. After 24 h cultivation, 25 ml of these cultures with small pellets were inoculated to 25 ml of the same medium with double concentration of all components (basal medium, glycerol and TNT). At the indicated time, 1 ml samples were taken from the flasks and after centrifugation, the enzyme activities were analyzed in the supernatants.

### Analytical methods

After *C. unicolor* cultivation in the glycerol-containing medium the fungal biomass was measured gravimetrically after recovering mycelium with centrifugation of whole cultures at 8000*g* for 20 min and drying at 70 °C for 24 h. When the fungus was cultivated in mandarin peels based medium the total nitrogen was determined according to Kjeldahl method with the Nessler reagent after pre-boiling of samples in 0.5 % solutions of trichloroacetic acid for 15 min to remove non-protein content. Protein was calculated as total N × 6.25.

Since, the mandarin peels contain various phenolic compounds interfering in analysis, the TNT content was determined only in the synthetic medium by a colorimetric assay (Jenkins 1990). One milliliter of sample was added to 1 ml of acetone; then 5–10 μl of 2 M KOH was added to make the solution alkaline and then 5 mg of sodium sulfite was added to stabilize the reaction product. After 5 min incubation at room temperature, the mixture’s A_462_ was read.

The laccase activity was determined spectrophotometrically (Camspec M501, UK) at 420 nm as the rate of 0.25 mM ABTS (2,2′-azino-bis-[3-ethyltiazoline-6-sulfonate]) oxidation in 50 mM Na-acetate buffer (pH 3.8) at room temperature (Bourbonnais and Paice 1990). MnP activity was measured at 270 nm by following the formation of a Mn^3+^-malonate-complex (Wariishi et al. 1992). One unit of laccase or MnP activity was defined as the amount of enzyme that oxidized 1 μmol of substrate per minute.

Microbiological experiments were performed twice using three replicates each time. All enzyme activity measurements were performed twice. Data presented correspond to the mean values with the standard deviations of the means shown as ± values.

## Results

### Effect of TNT on the enzyme production by the selected fungi

Six WRB strains have been selected to study the TNT effect on enzyme production in synthetic and lignocellulose-containing media. These fungi, with the exclusion of *P. coccineus*, usually express appreciable levels of the laccase and MnP activities in the mandarin peels-based medium (Elisashvili and Kachlishvili 2009). All fungi grew well in form of pellets in both media producing 3.2–4.3 g mycelial biomass/l in the presence of glycerol. Their growth accompanied with increase of the media pH from 5.8 to 6.0–7.3.

Detection of the fungi laccase activity revealed their capability to secrete this enzyme in the both tested media, although *P. coccineus* distinguished with low productivity in synthetic medium (Table 1). By contrast, *C. unicolor* BCC 300 followed by *T. versicolor* produced high levels of laccase in glycerol-based medium. Supplementation of this medium with 0.2 mM TNT provided more than twofold increase of laccase activity in cultures of *C. unicolor*, *P. coccineus*, and *T. versicolor*. However, the stimulation of the laccase production by TNT was not observed when *F. fomentarius* and *F. trogii* were cultivated in presence of this compound in the synthetic medium. As expected, substitution of glycerol in the nutrient medium with the mandarin peels as a growth substrate significantly promoted laccase expression by all fungi 3- to 45-fold increasing the enzyme activity. Especially both strains of *C. unicolor* were very potent laccase producers. The data obtained show the fungi differential response to the addition of 0.2 mM TNT to the mandarin peels-based medium. Enzyme production significantly strengthened in cultures of *C. unicolor* and *F. trogii*. Like the synthetic medium, no positive effect of TNT on the laccase synthesis was observed in fermentation of mandarin peels by *F. fomentarius*, *P. coccineus*, and *T. versicolor*.

**Table 1:** WRB enzyme activity and TNT removal in dependence on the media composition

Fungi	Media composition	Laccase (U/ml)	MnP (U/ml)	TNT removal (%)
*C. unicolor* BCC 300	Glycerol	15.6 ± 2.2^10a^	1.34 ± 0.2^5^	
	Glycerol + TNT	38.1 ± 5.2^10^	1.36 ± 0.2^7^	95 ± 2
	Mandarin peels	96.3 ± 13.3^14^	2.42 ± 0.4^7^	
	Mandarin peels + TNT	206.3 ± 35.0^14^	2.29 ± 0.4^7^	ND
*C. unicolor* BCC 302	Glycerol	3.7 ± 0.4^14^	0.63 ± 0.1^7^	
	Glycerol + TNT	10.7 ± 1.6^10^	0.78 ± 0.1^10^	96 ± 3
	Mandarin peels	80.2 ± 12.0^14^	1.56 ± 0.3^14^	
	Mandarin peels + TNT	121.8 ± 19.2^7^	1.95 ± 0.4^10^	ND
*F. fomentarius* BCC 38	Glycerol	5.5 ± 0.7^14^	0	
	Glycerol + TNT	5.3 ± 1.0^10^	0	66 ± 3
	MP	16.2 ± 2.5^10^	1.06 ± 0.2^10^	
	MP + TNT	19.2 ± 3.6^14^	1.24 ± 0.2^10^	ND
*F. trogii* BCC 146	Glycerol	3.5 ± 0.5^14^	0	
	Glycerol + TNT	4.2 ± 0.5^14^	0	70 ± 3
	MP	14.0 ± 2.0^14^	0.17 ± 0.03^10^	
	MP + TNT	30.4 ± 4.8^14^	0.15 ± 0.03^10^	ND
*P. coccineus* BCC 310	Glycerol	0.1 ± 0.02^5^	0	
	Glycerol + TNT	0.7 ± 0.1^7^	0	27 ± 2
	MP	4.5 ± 0.7^7^	0	
	MP + TNT	3.6 ± 0.7^7^	0	ND
*T. versicolor* BCC 775	Glycerol	7.6 ± 1.0^7^	0.14 ± 0.02^5^	
	Glycerol + TNT	13.6 ± 2.1^7^	0.11 ± 0.02^5^	92 ± 3
	MP	21.6 ± 3.3^14^	0.34 ± 0.05^14^	
	MP + TNT	17.8 ± 3.5^7^	0.45 ± 0.09^5^	ND

^a^The numbers indicate the days of the peak activity. 0.2 mM TNT was added to the glycerol and mandarin peels containing media before inoculation

As it was predicted, no MnP was detected in *P. coccineus* cultivation in the synthetic or lignocellulose-containing media. However, *F. fomentarius* and *F. trogii* also did not secrete this enzyme in the submerged cultivation in glycerol-based synthetic medium. By contrast, both strains of *C. unicolor* appeared to be efficient producers of MnP in the same cultivation conditions accumulating 0.63–1.34 U/ml of enzyme activity. Practically no stimulating effect of TNT on the enzyme expression was revealed in these and other cultures grown in synthetic medium. The mandarin peels provided a considerable increase of the MnP activity in all of studied fungal cultures capable to produce this enzyme increasing 2- to 3-fold *C. unicolor* and *T. versicolor’*s enzyme activity. It is noteworthy that in the cultures of *F. fomentarius* and *F. trogii* induction of MnP synthesis was revealed. However, supplementation of 0.2 mM TNT in this medium did not significantly affect the tested fungi MnP production.

At the end of cultivation (after 14 days), the TNT content was measured in the tested fungi culture liquids. The data in Table 1 indicate that both strains of *C. unicolor* and *T. versicolor* appeared to be the most appropriate fungi for the xenobiotic elimination. *F. trogii* and *F. fomentarius* also caused extensive TNT degradation, while *P. coccineus* inefficiently removed this compound.

### Effect of the TNT concentration

In this study, the TNT concentrations up to 0.4 mM were tested to determine their effect on the laccase and MnP production in the submerged fermentation of mandarin peels with the best enzyme producer and TNT degrader, *C. unicolor* BCC 300. It is worth noting that in the medium with the highest concentration of xenobiotic, the fungus mycelial pellets formation was delayed for 3 days. However, the maximum levels of the biomass accumulation were practically the same in all media after 10 and 14 days of the fermentation (data not shown).

Increasing of the TNT concentration from 0 to 0.4 mM resulted in considerable increase in laccase activity, the highest value being 240.7 U/ml at 0.4 mM TNT (Table 2). However, the profiles of laccase accumulation significantly differed in dependence on the aromatic compound concentration. In particular, the data received show that elevation of TNT concentration from 0 to 0.2 mM accelerated the laccase production during the first 5 days cultivation. When the TNT content was increased to 0.4 mM a significant reduction in the laccase activity was observed due to toxic effect on the fungus growth and after 5 days it was more than 2- to 3-fold lower as compared with those in the control and 0.2 mM TNT-containing media. In the following days, the fungal culture overcome the xenobiotic toxic effect and the level of secreted laccase in 0.4 mM TNT-containing medium appeared higher than in the other media. Another picture was revealed in the MnP activity measurement. Supplementation of nutrient medium with TNT did not favor MnP secretion and accumulation of this enzyme activity. On the contrary, even 0.2 mM TNT delayed MnP secretion during first 5 days fermentation while enzyme activity was not detected in medium containing 0.4 mM TNT. Hence, the MnP biosynthesis is more sensitive to the toxic action of TNT. It is worth noting that during the fungus subsequent cultivation the enzyme expression was triggered and the active secretion of MnP was occurred.

**Table 2:** Effect of the TNT concentration on the *C. unicolor* BCC 300 laccase and MnP production in the mandarin peels submerged fermentation

TNT (mM)	Laccase (U/ml)				MnP (U/ml)			
	Days of cultivation							
	5	7	10	14	5	7	10	14
0	36.8 ± 3.1	76.8 ± 8.7	89.4 ± 8.1	92.4 ± 10.3	0.91 ± 0.1	1.98 ± 0.3	2.31 ± 0.3	1.48 ± 0.2
0.1	43.0 ± 4.5	108.2 ± 13.1	127.5 ± 14.8	139.4 ± 21.0	0.93 ± 0.2	1.63 ± 0.3	2.49 ± 0.4	1.79 ± 0.3
0.2	50.4 ± 7.0	122.0 ± 20.1	182.0 ± 23.0	197.2 ± 33.6	0.62 ± 0.1	1.51 ± 0.3	2.30 ± 0.3	1.87 ± 0.3
0.4	15.5 ± 2.8	91.7 ± 18.1	173.4 ± 22.3	240.7 ± 34.2	0	0.49 ± 0.1	1.24 ± 0.2	1.81 ± 0.3

**Fig. 1:**
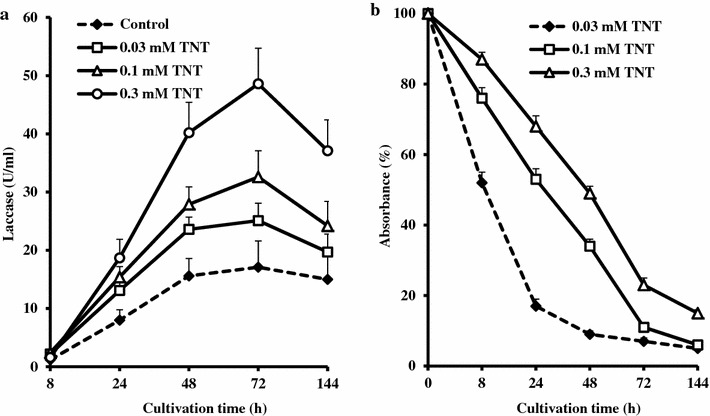
Kinetics of *C. unicolor* BCC 300 laccase activity (**a**) and TNT removal (**b**) in dependence on the xenobiotics concentration in the glycerol-containing medium

When *C. unicolor* BCC 300 was cultivated in the glycerol-based medium the fungus initial growth was significantly reduced in the medium containing 0.3 mM TNT (Table 3). Obviously this concentration had a detrimental effect on the organism because of toxic effect of xenobiotic and the fungus biomass level after 2 days cultivation in the presence of 0.3 mM TNT was threefold lower as compared to the control medium. Nevertheless, after 6 days of the submerged cultivation the fungus biomass reached the same values in all media. MnP activity appeared after 24 h in all variants of experiment; the enzyme activity gradually increased and achieved approximately the same values at the end of cultivation. The lower concentrations of TNT did not interfere in the MnP secretion by *C. unicolor* while at the highest concentration of this compound the enzyme activity after 2 days of fungus cultivation was three times lower than it was in other media. However, the enzyme production was not delayed since the fungus productivity for MnP after 48 h cultivation in the presence of 0.3 mM TNT (0.67 U/mg biomass) was as high as it was in the control medium (0.62 U/mg biomass). Unlike MnP, the *C. unicolor* laccase activity was detected already after 8 h of growth and practically no delay in the enzyme secretion was observed (Fig. 1a). Moreover, the higher was the TNT concentration the higher acceleration of laccase production and enzyme activity was observed after 72 h of fungus cultivation. Almost threefold increase of the laccase activity was achieved in the fungus cultivation in presence of 0.3 mM TNT (48.6 U/ml) as compared with the control medium (17.1 U/ml). An assay of TNT content in culture medium revealed the gradual decrease of the xenobiotic concentration till the end of experiment (Fig. 1b). After the 6 days of *C. unicolor* cultivation in media containing 0.1 and 0.3 mM TNT, respectively, only 6 and 15 % of the initial TNT content was detected.

**Table 3:** Effect of the TNT concentration on the *C. unicolor* BCC 300 growth and MnP activity in short-term cultivation in the glycerol-based medium

TNT (mM)	Biomass gain (mg/ml)		MnP (U/ml)			
	Days of cultivation					
	2	6	1	2	3	6
0	1.2 ± 0.1	3.2 ± 0.2	0.23 ± 0.03	1.43 ± 0.1	2.08 ± 0.3	2.37 ± 0.3
0.03	1.3 ± 0.1	3.3 ± 0.2	0.24 ± 0.03	1.55 ± 0.2	2.06 ± 0.3	2.42 ± 0.4
0.1	1.1 ± 0.1	3.2 ± 0.2	0.25 ± 0.04	1.51 ± 0.3	1.95 ± 0.3	2.55 ± 0.5
0.3	0.4 ± 0.1	3.2 ± 0.2	0.07 ± 0.01	1.01 ± 0.2	1.54 ± 0.3	2.29 ± 0.4

## Discussion

In the present study, the LME activity of the five WRB species has been found to be highly influenced with growth substrate and TNT. Glycerol and mandarin peels have been used as cheap and best carbon and energy sources to provide the fungi growth and target enzymes production and as co-substrates for TNT transformation. It is worth noting that in the fungi cultivation in the medium without glycerol or mandarin peels, 0.2 mM TNT didn’t serve as the sole carbon source and not to growth of fungi was observed (data not shown). Other authors also used co-substrates like mono-, disaccharides, sugar alcohols, organic acids, and molasses to provide TNT transformation (Stahl and Aust 1993; Boopathy et al. 1994; Rho et al. 2001; Cheong et al. 2006).

Some conclusions can be drawn from the data obtained in this study. Firstly, these data clearly indicate that the tested fungal strains belonging to different taxonomic groups display a wide diversity in their response to growth substrate in the nutrient medium (Table 1). When the WRB cultivation was conducted in the glycerol-containing medium, *P. coccineus* BCC 310, like *Ganoderma* spp. and *Pleurotus tuber*-*regium* (Elisashvili and Kachlishvili 2009), secreted very low laccase activity, whereas other fungal strains produced significant laccase activity ranging from 3.5 U/ml (*F. trogii*) to 15.6 U/ml (*C. unicolor* BCC 300) in the same synthetic medium. In the other studies, *C. unicolor* C-139 accumulated 1.1 U/ml (Janusz et al. 2007) and 1.7 U/ml (Rola et al. 2013) laccase in the synthetic media with glucose and maltose, respectively. Secondly, only the *C. unicolor* strains and with lesser extent *T. versicolor* BCC 775 were capable to express MnP activity in the synthetic medium. By contrast, Michniewicz et al. (2006) showed that *C. unicolor* C-137 did not secrete the peroxidase activity in both glucose-containing synthetic and in complex tomato juice-based media. However, our finding is in a good agreement with observations of other researchers. For example, Hibi et al. (2012) showed that *Cerrena* sp. was capable of secreting laccase and three peroxidases in submerged cultivation in a glucose-containing medium. Thirdly, the present study highlights the role of growth substrate in the LME activity expression. Replacement of glycerol with mandarin peels promoted the MnP secretion by these fungi and manifold increased the laccase activity in all tested WRB. More interesting is that the mandarin peels containing medium provided induction of the MnP synthesis by *F. fomentarius* BCC 38 and *F. trogii* BCC 146 suggesting that the presence of the lignocellulose in the nutrient medium is a prerequisite for this enzyme production by these fungi. In this respect, our results are in agreement to the earlier findings reporting that MnP activity was lacking during cultivation of fungi in the synthetic media and appeared only during growth in the presence of plant materials (Schlosser et al. 1997; Kapich et al. 2004; Elisashvili et al. 2006). Kapich et al. (2004) proved that the presence of extractive aromatic/phenolic substances, derived from straw, was essential for the MnP production by *P. chrysosporium* in submerged cultures. A number of other reports indicate that aromatic compounds, especially structurally related to lignin, play an important role in regulation of the LME production in basidiomycetous fungi (Elisashvili and Kachlishvili 2009; Bertrand et al. 2013). Finally, unlike many other WRB, *C. unicolor* BCC 300 has ability to synthesize high levels of LME under high carbon and high nitrogen conditions and it secretes high laccase (Fig. 1) and MnP (Table 3) activities during trophophase as it has been shown in the short-term experiments. Undoubtedly, this strain of *C. unicolor* accumulating in the shake-flasks experiments as high as 240 U laccase/ml and simultaneously 2 U MnP/ml is a good candidate for scaling up LME production in the laboratory fermenter.

The data received in this study show that with the exclusion of *F. fomentarius* BCC 38 and *F. trogii* BCC 146 laccase formation by other fungi was strongly affected with TNT. Laccase productivity of the *C. unicolor* strains, *P. coccineus*, and *T. versicolor* increased 2.4–2.9-, 7-, and 1.8-fold, respectively, when 0.2 mM TNT was supplemented in the synthetic medium. These results suggest that enhancement of laccase production in response to TNT differs greatly among the different WRB. However, the TNT supplementation to the nutrient medium failed to increase the MnP activity. The reason for differential effect of TNT is not obvious, but the most likely explanation is that, this compound or some TNT transformation metabolites specifically trigger laccase production by *C. unicolor* BCC 300 and other fungi to eliminate their toxic action. Finally, stimulation of laccase production by this fungus with TNT is concentration-dependent since the activity of the secreted enzyme gradually increased with an elevation of TNT concentration. In particular, supplementation of 0.2 and 0.4 mM TNT in the mandarin peels-based medium increased of the laccase activity more than twofold as compared to it was in the control medium (Table 2). When *C. unicolor* BCC 300 was cultivated in the synthetic medium, addition of 0.03, 0.1, and 0.3 mM TNT did not affect the fungus biomass level and the MnP activity (Table 3), but specifically stimulated the laccase production (Fig. 1) and increased the fungus productivity from 6.8 to 9.8, 12.7 and 26.8 U/mg biomass, respectively. Recently, it has been shown that 0.3 mM TNT became toxic to *C. unicolor* grown in the mannitol-containing medium; however, at this exact concentration of xenobiotic the highest differential rate of laccase synthesis was observed (Elisashvili et al. 2010). In this study, 0.3 mM TNT only delayed the fungus growth in the glycerol-containing medium during the first day of the submerged cultivation and provided the highest laccase activity since 3 days of growth. The mechanism which TNT or the TNT metabolites specifically affect the laccase production by certain fungi, in particular by *C. unicolor* BCC 300, is unclear. We assume that the sensitivity of these fungi to TNT and chemical stress are responsible for such a response to the xenobiotic presence. It should be noted that Stahl and Aust (1993) pointed out that the extracellular detoxification of TNT can be maximized at starting with significant amounts of *P. chrysosporium* mycelium. Taking into account this circumstance, TNT was fed to the immobilized *P. chrysosporium* only after the 7th day of growth period (Rho et al. 2001). Moreover, TNT and its catabolites were added only to the 5-day-old culture of *T. versicolor* since the fungus could not grow when these compounds have been supplemented to the medium at inoculation time (Cheong et al. 2006). In this study, *C. unicolor* and other fungi were capable to survive at comparatively high concentrations of TNT supplemented to the media at the time of inoculation and could efficiently produce the LME.

There are few reports on the TNT biodegradation with the white-rot and litter-decaying basidiomycetes. Successful degradation and significant mineralization of this recalcitrant compound was achieved with *P. chrysosporium* and other WRB (Fernando et al. 1990; Van Aken et al. 1999; Kim and Song 2000; Cheong et al. 2006). Usually the LME are considered to be implicated in the degradation of TNT and its catabolites because many these enzymes genes are expressed in presence of the diverse recalcitrant organic pollutants. In particular, Van Aken et al. (1997, 1999) showed that a concentrated preparation of MnP from *Phlebia radiata* was able to transform TNT (22 % mineralization) and 2-amino-4, 6-dinitrotoluene (76 % mineralization). However, none of the earlier studies evaluated the TNT effect on the LME production. Cheong et al. (2006) determined the laccase gene expression by *T. versicolor* during degradation of TNT and established that the expression levels of the laccase gene in cultures containing TNT and its catabolites were fourfold higher than that in the control culture. The increase of the gene expression was the most prominent at the early phase of addition (6 h) and the laccase gene from this fungus was considered to be implicated in the degradation of xenobiotic. Our results are in agreement with this conclusion. In this study, a direct demonstration of the TNT mineralization by the tested fungi was not performed. However, among them, two strains of *C. unicolor* followed by *T. versicolor* BCC 775 produced high levels of laccase in addition to the appreciated MnP activity and almost completely removed TNT from the synthetic medium (Table 1). It is not excluded that the presence of both enzymes provided a cooperative effect on the TNT removal. Two fungi, *F. fomentarius* BCC 38 and *F. trogii* BCC 146, also secreted the high laccase activity, but MnP was not produced. Obviously, this circumstance explains their moderate efficiency in the TNT elimination. Finally, *P. coccineus* BCC 310 did not produced MnP and expressed comparatively low laccase activity in the synthetic medium and, respectively, this fungus removed only 27 % from the initial TNT content. In the short-term experiments, a certain reduction in the TNT concentration was observed after initial 8 h cultivation likely due to the compound adsorption by the fungal mycelium and action of enzymes present in the inoculum (Fig. 1). However, during the next days, laccase and MnP contributed in the TNT significant disappearance. The data have shown a correlation between the TNT removal and laccase and especially MnP activity of the tested fungi. Nevertheless, a fundamental work with the isolated enzyme preparations having different ratio of individual LME and purified enzymes are needed to establish their role in the TNT mineralization chain. It is obviously that there are the other enzymes and factors governing degradation of this compound which should be established.

Thus, this study has shown a high applicability of *C. unicolor* BCC 300 not only for the LME production, but also for the TNT and other xenobiotics biodegradation. Our finding that this strain has been resistant to the high concentration of TNT (0.3 mM in glycerol-containing medium and 0.4 mM in mandarin peels-based medium) and has ability to produce in such environmental conditions high levels of extracellular laccase and MnP is important for a rapid and efficient bioremediation of the TNT polluted areas.

## Conclusions

Six strains of WRB belonging to the different taxonomic groups exerted a wide diversity in their response to carbon source and the TNT supplementation to the nutrient medium. Firstly, while all tested fungi were capable to produce laccase in both glycerol and mandarin peels-containing media, only *C. unicolor* BCC 300 and *T. versicolor* BCC 775 secreted MnP in the glycerol-containing medium. Secondly, replacement of glycerol by mandarin peels leaded to increase of the fungi laccase activity, promoted *C. unicolor* and *T. versicolor* MnP secretion and induced this enzyme production by *F. fomentarius* BCC 38 and *F. trogii* BCC 146. Thirdly, differential response of the WRB strains to the TNT addition also was observed. In particular, *C. unicolor* BCC 300 laccase activity increased 2- to 3-fold in both media whereas the stimulation of laccase production was not revealed in cultivation of *F. fomentarius* BCC 38. Moreover, TNT practically did not affect the MnP secretion. Fourthly, while two strains of *C. unicolor* followed by *T. versicolor* BCC 775 almost completely removed 0.2 mM TNT from the synthetic medium, *P. coccineus* BCC 310 degraded only 27 % of xenobiotic indicating involvement of MnP in this process. Finally, this study revealed *C. unicolor* BCC 300 as an appropriate candidate for the LME production and TNT biodegradation. This strain tolerates high concentration of toxic compound and has been capable to synthesis at such conditions high titers of the target enzymes.
